# Investigation of the Presence of Biofilms in Chronic Suppurative Otitis Media, Nonsuppurative Otitis Media, and Chronic Otitis Media with Cholesteatoma by Scanning Electron Microscopy

**DOI:** 10.1155/2013/638715

**Published:** 2013-10-27

**Authors:** Ercan Kaya, Ilknur Dag, Armagan Incesulu, Melek Kezban Gurbuz, Mustafa Acar, Leman Birdane

**Affiliations:** ^1^Department of Otorhinolaryngology, Medical Faculty, Eskisehir Osmangazi University, School of Medicine, 26480 Eskisehir, Turkey; ^2^Electron Microscope Laboratory, Eskisehir Osmangazi University, 26480 Eskisehir, Turkey; ^3^Department of Otorhinolaryngology, Yunusemre State Hospital, 26190 Eskisehir, Turkey

## Abstract

*Objective*. Biofilms have been shown to play a major role in the pathogenesis of otolaryngologic infections. However, very limited studies have been undertaken to demonstrate the presence of biofilms in tissues from patients with chronic otitis media (COM) with or without cholesteatoma. Our objective is to study the presence of biofilms in humans with chronic suppurative and nonsuppurative otitis media and cholesteatoma. *Study Design*. In all, 102 tissue specimens (middle ear, mastoid tissue, and ossicle samples) were collected during surgery from 34 patients. *Methods*. The samples were processed for the investigation of biofilms by scanning electron microscopy (SEM). *Results*. Our research supports the hypothesis in which biofilms are involved in chronic suppurative otitis media, cholesteatoma, and, to a lesser degree, chronic nonsuppurative otitis media. There were higher rates in hypertrophic and granulated tissue samples than in normal mucosa. In addition, the presence of biofilms was significantly higher in the middle ear mucosa compared with the mastoid and ossicle samples. *Conclusion*. In the clinic, the careful use of topical or systemic antimicrobials is essential, and, during surgery, hypertrophic tissue must be carefully removed from normal tissue.

## 1. Introduction

Biofilms are complex bacterial communities that adhere to the surface of implanted biomaterial or mucosa [1]. They are embedded in a slim-like extracellular matrix composed of proteins, polysaccharides, and nucleic acids known as extracellular polymeric substances (EPS) [2]. Because they have effective defense mechanisms against the immune system of their host and against antimicrobial agents, they are difficult to eradicate [3, 4]. 

Today, the diagnosis, treatment, and prevention of biofilm infections clearly require different strategies from those used against acute infections [5]. In particular for mucosal biofilms, we need to better understand the interaction between the bacterial attachment and the human host [6]. The altered microenvironment in the mucosa and the degree of colonization are also important [7]. 

The importance of biofilms in otolaryngologic infections is becoming increasingly apparent [8]. At present, much of the literature on this subject involves in vitro studies, with the majority related to complications involving medical implants [9]. Recently, a number of publications have shown the presence of biofilms on the mucosal surfaces of tonsils and adenoids. Biofilm has also been demonstrated in otitis media with effusion and direct biopsy specimens of the middle ear mucosa and in a nonhuman primate model of chronic otitis media [10]. Biofilms are nearly impossible to detect with standard culture techniques [11] because these techniques do not elucidate the complex, three-dimensional aspects of biofilms. Molecular diagnostics based nucleic acid upon amplification strategies have provided the means to be detected and identify bacteria, and various imaging technologies have given researchers insights into the role of biofilms in human infections [12]. Scanning electron microscopy (SEM) is also an advanced resolution method that provides ultrastructure analysis of biofilms [13]. Our objective is to study the presence of biofilm in humans with chronic otitis media with or without cholesteatoma. 

## 2. Materials and Methods

Patients undergoing surgical treatment were asked to participate in our study. The study was approved by the ethics committee of the Faculty of Medicine of Eskisehir Osmangazi University. The tissue samples were collected during routine surgical treatment from 34 patients in the Eskisehir Osmangazi University Medical Faculty during the period between October 2011 and May 2012. These patients included 16 females and 18 males. The chronic otitis media (COM) patients were divided into three groups: chronic suppurative otitis media (CSOM) (*n* = 10, 30 specimens); chronic nonsuppurative otitis media (CNSOM) (*n* = 11, 33 specimens); and chronic otitis media with cholesteatoma (*n* = 13, 39 specimens). Various tissue samples from the patients in each group were harvested including from the middle ear mucosa, mastoid tissue, and ossicle. In addition, during the surgery, the middle ear mucosa was classified as normal, hypertrophic, or granulated tissue with associated mucosa. Tissue was taken only if the debridement of the tissue was necessary during the surgical treatment. Any eroded ossicle that could not be used for reconstruction was also removed and evaluated for biofilm formation.

Our cases with cholesteatoma represented acquired cholesteatoma cases, and they were divided into three groups according to the location of the tissue: attic (A), sinus (S), and pars tensa (PT) [14]. 

The tissue samples were immediately placed in 2.5% glutaraldehyde (prepared in 0.1 M phosphate buffer, pH 7.4) for 24 hours at 4°C as a prefixation step. They were then rinsed twice with 0.1 M phosphate buffer (pH 7.4), postfixed using 1% osmium tetroxide for 1 hour at room temperature, and finally rinsed with distilled water. Next, the specimens were dehydrated using graduated concentrations of ethyl alcohol (30%, 50%, 70%, 90%, and 96%) for 15 minutes each followed by absolute alcohol for 30 minutes. The specimen was dried using the critical point dryer Polaron CPD 7501 Critical Point Dryer (VG. Microtech, East Sussex, UK). For mounting, carbon conductive paint was used; for specimens, gold coating with Polaron SC7620 Sputter Coater was used. Finally, each specimen was examined using a JEOL scanning electron microscope (JEOL JSM-5600LV). Several areas of each sample were systematically scanned. A sample was considered to have a biofilm if 3 criteria were met: (1) presence of bacterial-sized and -shaped objects; (2) presence of an amorphous material, consistent with glycocalyx around the bacteria; and (3) surface binding [15, 16]. 

## 3. Results

A total of 102 specimens were collected from 34 patients. The mean age of patients was 40.8 years for the CSOM group, 34.7 ± 11.6 years for the CNSOM group, and 28 ± 23.7 years for the cholesteatoma group. Of the 10 CSOM patients, biofilm formation was observed in 7 (70%) cases by SEM. In the CNSOM group, 6 of 11 (54.5%) patients showed a biofilm. Eight (61.5%) of the 13 patients with cholesteatoma had a biofilm (Table 1). The biofilm findings according to the specimen distribution were presented in Table 1. 

Among tissue samples obtained from the three-patient groups, biofilm formation was the most frequently observed in the middle ear mucosa samples (50% in CSOM group, 54.5% in the CNSOM group, and 38.4% in the cholesteatoma group). During the surgery, intraoperative cases of biofilm-positive samples were evaluated, with the results presented in Table 2. We found that the biofilm rate was higher in hypertrophic and granulated tissue than in normal mucosa. In the cholesteatoma cases, the biofilm conditions depending on the location are presented in Table 3. Additionally, because the number of biofilm-positive samples was low in this group, whether the biofilm shows a significant difference depending on the location of the cholesteatoma could not be determined.

**Figure 1:**
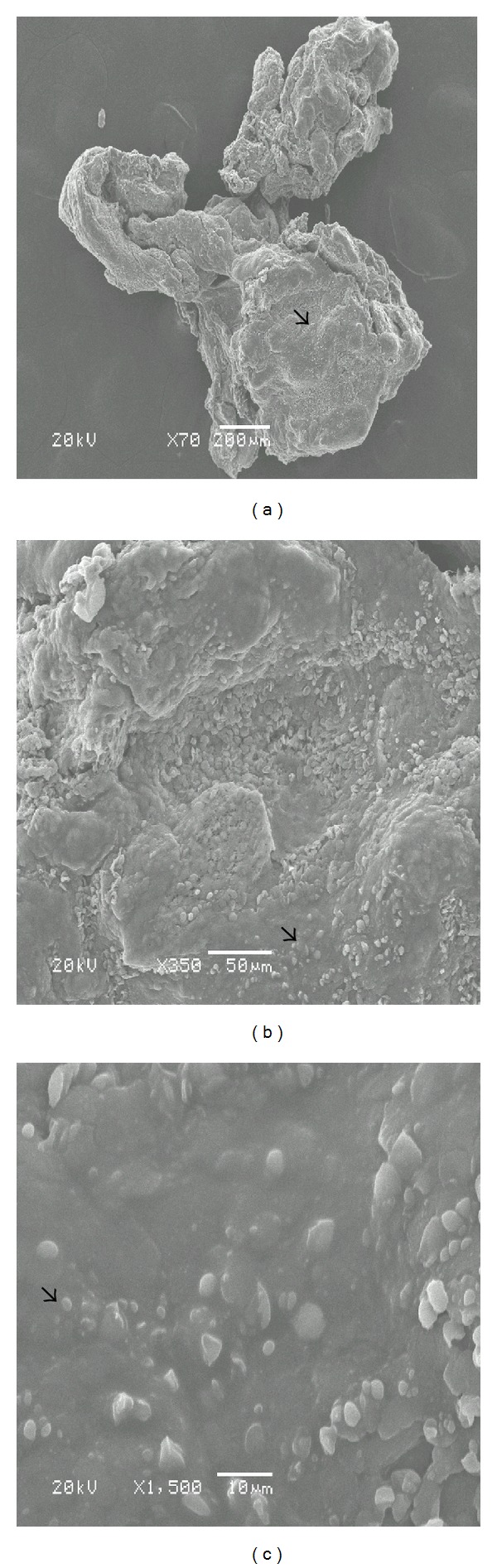
(a) Scanning electron micrograph of middle ear tissue covered with biofilm. Arrows indicate the extracellular material connected to the bacteria. The specimen was removed from a patient undergoing surgery for nonsuppurative chronic otitis media ((b) and (c) higher magnifications of same pictures).

**Figure 2:**
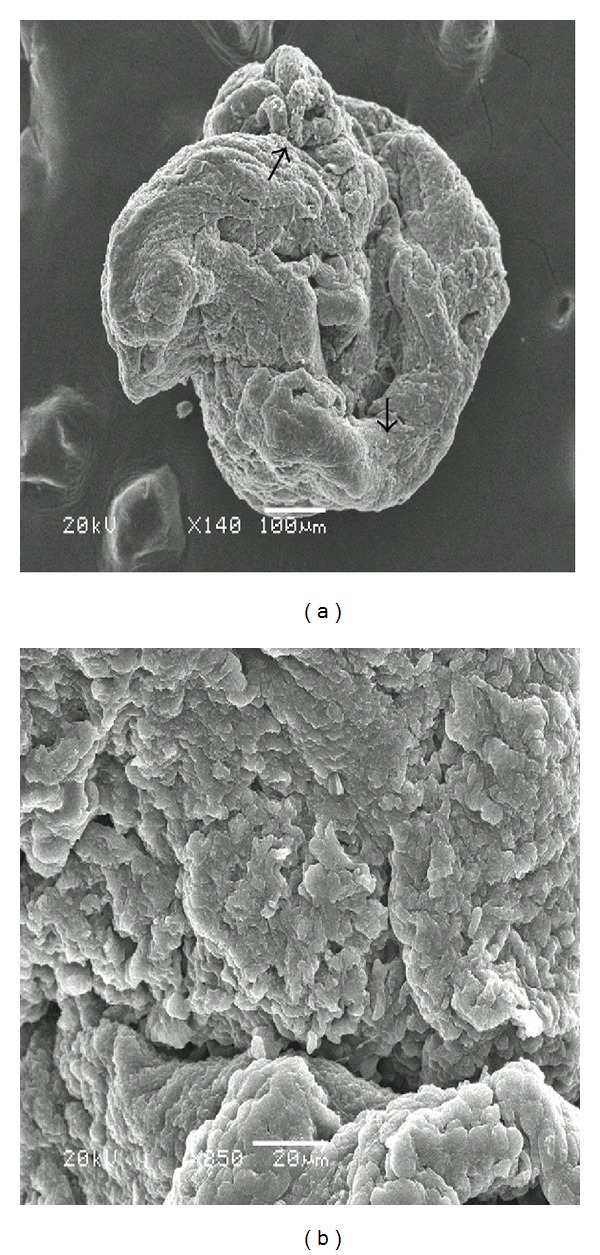
(a) Arrows indicated that the biofilm suspected regions; (b) however, in some samples, rough surface structure was seen as the magnification increased.

**Figure 3:**
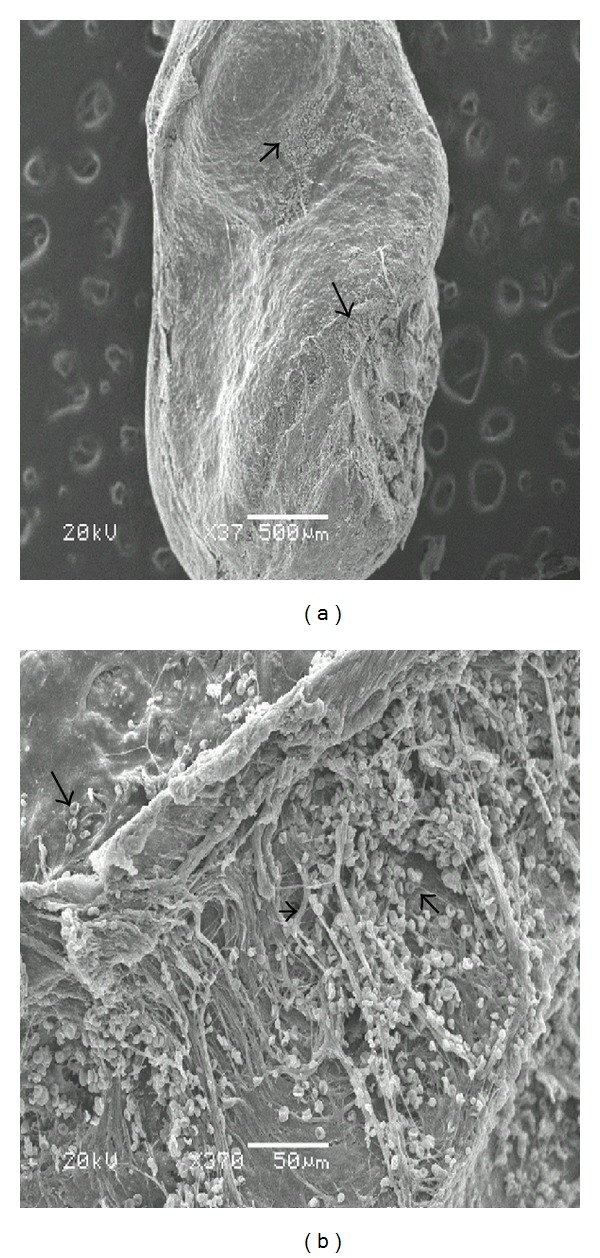
This image shows a middle ear sample surface. Specimen was taken from a patient undergoing surgery for chronic otitis media with cholesteatoma. This sample appeared to be biofilm positive showed at low magnification, but erythrocytes (arrows) were seen as magnification increased.

**Figure 4:**
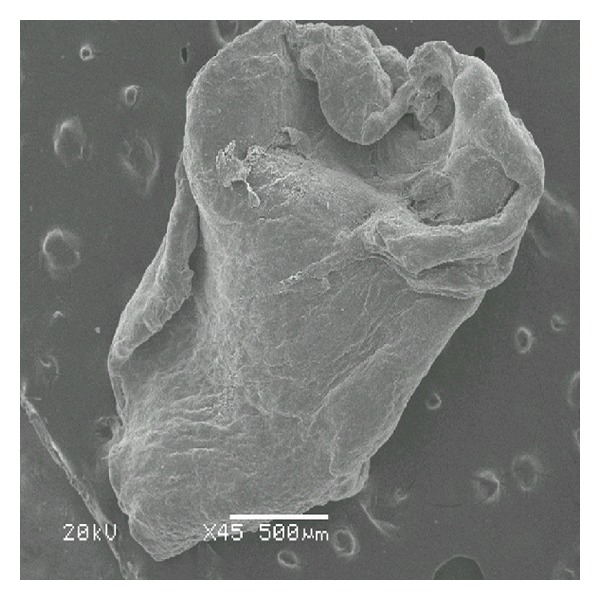
This image shows the surface of a middle ear of a patient with chronic suppurative otitis media. The specimen was used as a control in our study. Note the relatively smooth surface and lack of organisms.

Scanning electron microscopy demonstrated that the distribution of bacterial microcolonies was not homogenous throughout the tissue surface in biofilm-positive samples. In some areas, extracellular material was observed connecting the bacteria (Figures 1(a), 1(b), and 1(c)). Occasionally, in those samples that appeared to be negative at low magnifications, the presence of a biofilm was encountered as the magnification increased. In contrast, occasionally, samples that appeared to be positive at low magnifications showed, a rough surface structure of the tissue (Figures 2(a) and 2(b)) or erythrocytes as the magnification increased (Figures 3(a) and 3(b)).  Figure 4 also indicated that the biofilm negative sample. 

## 4. Discussion

The data presented in this study support the hypothesis that biofilms may play a significant role in otolaryngologic infections. In particular, the greater presence in patients with CSOM (7 of 10, 70%) and cholesteatoma (8 of 13, 61.5%) does suggest that the biofilms are pathogenically important. With respect to this correlation, Lee et al. [17] reported that frequency of biofilms was 60% (6 of 10) in CSOM, and Lampikoski et al. [18] reported 66% (19 of 29) in mastoid mucosa with CSOM. Therefore, Roland proposed that biofilms are the likely cause of CSOM, which would explain the observed resistance to antibiotic therapy [19]. Biofilms may attach to damaged tissue, such as ulcerated middle ear mucosa or exposed osteitic bone, and are thought to cause persistent infections [20]. In addition, the frequent and inappropriate use of topical antibiotics and antiseptic solutions in COM may create a suitable environment for microorganism resistance.

As expected, we found the presence of biofilms to be significantly higher in patients with CSOM (70%) compared with those with CNSOM (54.5%). To our knowledge, there have not been any published data regarding these two groups and biofilm conditions that can be compared with our results. Thus, these findings warrant further investigation to determine the exact role of biofilms in the pathogenesis of CSOM and CNSOM infections. Recently, the pathogenesis of acquired cholesteatoma disease has been studied extensively, but the mechanisms are not yet fully understood. Lampikoski et al. reported biofilm formation in three of four infected cholesteatoma patients and in three of five (60%) cholesteatoma cases [18]. In our study, we found results similar to those from the literature (8 of 13, 61.5%). Lampikoski et al. indicated that the cholesteatoma tissue could be hypothesized to be a beneficial substrate for biofilms to settle upon [18]. Chole and Faddis described the presence of biofilms in human and gerbil cholesteatomas and identified biofilms in 16 of 24 clinical cases (66%) [21]. The authors suggested that the bacteria can infect the keratin matrix, forming biofilms that, in turn, lead to chronic persistent infections. In our study, cholesteatoma also appeared to be an ideal environment for the development of biofilms. 

Generally, the first choice for ossicle chain reconstruction in COM is to use the patient's own ossicles [22]. However, there is a risk of cholesteatoma matrix remaining on the ossicle in patients with cholesteatoma. Therefore, the use of the ossicles in reconstruction could be argued. 

According to the results of our study, the presence of biofilms was significantly higher in the middle ear mucosa compared with the mastoid and ossicle samples, likely because of the location of the middle ear mucosa near the external auditory canal. In addition, we determined that biofilm formation occurred less often in the ossicle samples. As known, the ossicles hang suspended inside of the tympanic cavity, and they have a relatively poor immune response. For this reason, in fact, we expected to find a higher biofilm rate in this region. However, in our study, ossicles were the locations on which the least biofilm was observed. Nevertheless, we did not observe a disruption depending on infection on the ossicle surfaces. This condition may also help prevent biofilm adhesion. 

The granulated tissue may be produced as a response to microbial biofilm adhesion to alloplastic materials such as tympanostomy tubes and partial or total ossicular replacement prosthesis or as a secondary consequence of bacterially induced inflammation in the middle ear. Chole and Faddis reported that recurrent infections or hypertrophy raises the possibility that the bacteria are sequestered from the host defenses [21]. In addition, hypertrophy is thought to be caused by multiple and sometimes resistant bacteria. In our study, we also determined that the biofilm rates were higher in hypertrophic and granulated tissue samples than in normal mucosa. 

A limitation of the present study is the lack of a control group. Tissue from an appropriate control group is ethically problematic to obtain because it should be composed of tissue from age-matched control subjects who have never had an infection of the upper airways. Thus, the inclusion of controls was not feasible in our study. 

Although SEM has been widely used by investigators to identify and characterize biofilms, we have experienced some drawbacks in using this method. For example, although our sample size is too small, surveying the entire specimen for biofilm detection was difficult. Occasionally, because of the rough topographic structure of the surface or crypts, these regions could not be examined in detail. Recently, newer techniques, such as confocal laser scanning microscopy, have also been used in biofilm research. These methods allow for further elucidation of the structure-function relationships in biofilms. However, we were unable to find any studies in the literature comparing the sensitivity and specificity of the microscopic techniques used to detect human host biofilms.

In conclusion, our research supports the hypothesis in which biofilms are involved in CSOM, cholesteatoma, and, to a lesser degree, CNSOM. In this situation, the careful use of topical or systemic antimicrobials is essential. The first choice is surgery, and, during the surgery, hypertrophic tissue must be carefully removed from the normal tissue. There are many reasons for failure after the operation. If the tissue with the potential to harbor biofilms, such as granulated tissue, cannot be cleaned sufficiently, residual biofilms may be one reason for the surgery failure.

## Figures and Tables

**Table 1:** The biofilm findings according to the specimen distribution in patient groups.

	CSOM				CNSOM				Cholesteatoma		
Pt	ME^a^	MAS^b^	OS^c^	Pt	ME	MAS	OS	Pt	ME	MAS	OS
1	−	+	−	1	+	−	−	1	+	−	−
2	+	−	−	2	−	−	−	2	−	−	−
3	−	−	−	3	+	+	−	3	+	−	−
4	−	+	+	4	+	+	−	4	−	−	+
5	−	−	−	5	+	+	−	5	+	−	−
6	+	+	+	6	−	−	−	6	+	−	−
7	+	−	−	7	−	−	−	7	−	−	−
8	−	−	−	8	+	−	+	8	−	+	−
9	+	−	+	9	−	−	−	9	−	−	−
10	+	−	−	10	+	−	+	10	−	+	−
				11	−	−	−	11	−	−	+
								12	+	−	−
								13	−	−	−

^a^Middle ear mucosa; ^b^mastoid mucosa; ^c^ossicle samples.

**Table 2:** The intraoperative condition of biofilm positive samples during the surgery.

Patient number	Diagnosis/condition	Description of specimen	The intraoperative condition of biofilm positive samples		
			Normal mucosa	Hypertrophic tissue	Granulation tissue with associated mucosa
10	Chronic suppurative otitis media	Middle ear mucosa	1 (20%)	1 (20%)	3 (60%)
		Mastoid tissue	1 (33.7%)	0	2 (67.3%)

11	Chronic nonsuppurative otitis media	Middle ear mucosa	1 (17%)	2 (33%)	3 (50%)
		Mastoid tissue	0	2 (67.3%)	1 (33.7%)

13	Cholesteatoma	Middle ear mucosa	0	2 (40%)	3 (60%)
		Mastoid tissue	0	0	2 (100%)

**Table 3:** The presence of biofilm in acquired cholesteatoma specimens.

Description of specimen	The number of biofilm positive samples		The number of biofilm negative samples	
Cholesteatoma middle ear mucosa	5 (38.4%)	A:3	8 (61.6%)	A:6
		S:1		S:1
		PT:1		PT:1

Cholesteatoma mastoid tissue	2 (15.3%)	A:2	11 (84.7%)	A:7
		S:0		S:2
		PT:0		PT:2

Cholesteatoma ossicle samples	2 (15.3%)	A:2	11 (84.7%)	A:7
		S:0		S:2
		PT:0		PT:2

A: attic; S: sinus tympani; PT: pars tensa.
